# Analysis of Forming Limits in Sheet Metal Forming with Pattern Recognition Methods. Part 1: Characterization of Onset of Necking and Expert Evaluation

**DOI:** 10.3390/ma11091495

**Published:** 2018-08-21

**Authors:** Emanuela Affronti, Christian Jaremenko, Marion Merklein, Andreas Maier

**Affiliations:** 1 Institute of Manufacturing Technology, Friedrich-Alexander-Universität Erlangen-Nürnberg Egerlandstr. 13, 91058 Erlangen, Germany; marion.merklein@fau.de; 2 Pattern Recognition Lab, Friedrich-Alexander-Universität Erlangen-Nürnberg Martensstr. 3, 91058 Erlangen, Germany; andreas.maier@fau.de

**Keywords:** forming limit curve, pattern recognition, sheet metal forming, machine learning

## Abstract

In automotive manufacturing, high strength materials, and aluminum alloys are widely used to address the requirement of ensuring a lightweight car body and correspondingly, reducing pollution. In this context of complexity of materials and structures, an optimized process design with finite element analyses (FEA) is mandatory, as well as a correct definition of the material forming limits. For this purpose, in sheet metal forming, the forming limit curve (FLC) is used. The FLC is defined by the onset of necking. The standard evaluation method according to DIN EN ISO 12004-2 is based on the cross-section method and assumes that the failure occurs due to a clear localized necking. However, this approach has its limitations, specifically in the case of brittle materials that do not exhibit a distinct necking phase. To overcome this challenge, a pattern recognition-based evaluation is proposed. Although pattern recognition and machine learning techniques have been widely employed in the medical field, few studies have investigated them in the context of analyzing metal sheet forming limits. The application of pattern recognition in metal forming is subject to the exact definition of the forming behaviors. Thereby, it is challenging to relate patterns on the strain distribution during Nakajima tests with the onset of necking for the FLC determination. Thus, the first approach was based on the crack evaluation, since this class is well-defined. However, of substantial interest is the evaluation of the general material instabilities that precede failure. Therefore, in the present study, the analysis of the material behavior during stretching is conducted in order to characterize instability classes. The results of Nakajima tests are investigated using an optical measurement system. A conventional pattern recognition approach based on texture features, considering the outcomes of expert interviews for the definition of classes is used for the FLC determination. Moreover, an analysis of the validity of the supervised learning is conducted. The results show a good prediction of the onset of necking, even for high strength materials with a recall of up to 92%. Some deviations are observed in the determination of the diffuse necking. The discrepancies of the different experts’ prognoses highlight the user-dependency of the FLC, suggesting further investigations with an data-driven approach, could be beneficial.

## 1. Introduction

The forming limit curve (FLC) is used in expecting the forming range of sheet metal without material instabilities. The limits are expressed in terms of major and minor strain pairs at necking. A comprehensive summary of the FLC history can be found in Col [1]. Nowadays the standard for the evaluation of the FLC is represented in Europe by the DIN EN ISO 12004-2 [2]. The stretching tests used are according to the Nakajima [3] and Marciniak [4] setups. The sheet metal is clamped in a clamping unit and a punch, hemispherical or flat for the Nakajima or Marciniak test respectively, forms the specimen due to its vertical stroke until fractures occurs. The evaluation method suggested in the standard is based on the Bragard’s study in 1972 [5] and it is known today as “cross-section” method. In this method, the strain distribution of the stage before crack is considered. Afterwards, sections perpendicular to the crack initiation are defined and the strain development on these sections is interpolated with a second-order polynomial, according to the common sense that the strain on such sections develops similar to a bell-shaped curve. In those years, the strain measurement techniques were based on putting circles patterns on the specimen surface before forming and measuring their diameter deformations after forming. However, it permitted only to identify the strain distribution on the last stage, without giving any information of the strain history.

In the 1990s, with the advent of the computer era, pictures could be digitalized during the deformation and analyzed with computer algorithms, based on digital image correlation techniques (DIC). The DIC correlates pictures of subsequent moments of the investigated test. Already in 1985 Chu et al. investigated the application of the DIC in experimental mechanics [6] and in 1999 it was used for the evaluation of the FLCs [7]. Due to using two cameras during Nakajima or Marciniak tests, the strain history on the surface can be measured. Although DIC allows an accurate and complete strain measurement, the standard method is still based only on the cross-section method. In addition, modern light materials such as high strength steels and aluminum alloys are characterized by multiple local strain maxima and sudden crack without necking phase. Consequently, for such materials the interpolation of the strain distribution with a second order function shows weaknesses [8].

To address this limitation, several methods that take into account strain history during Nakajima tests, have been proposed. The so-called “line-fit” method proposed by Volk et al. [9] or the correlation-coefficient method [10] are an example of “time-dependent methods”. They consider the thickness reduction in the instability zone and evaluate the onset of necking as a sudden change in the thickness reduction. In particular, the line-fit method has shown promising results in good agreement with the experiments. Nevertheless, the time-dependent methods take into account the necking area and thus still focus the attention only on the instability zone.

In 2015 [11] a new approach based on pattern recognition was proposed. The idea was to use machine learning for the pattern detection on Nakajima tests to incorporate new knowledge about the forming development. Conventional pattern recognition deals with the mathematical and technical aspects of automatic processing and evaluation of patterns [12], where a physical signal e.g., images or speech are converted into appropriate compact characteristic representations. In a supervised pattern recognition setup these characteristic representations are often related e.g., to the visual perception of experts that have an excellent understanding of the underlying problem to be solved. These experts annotate the data to be associated with a certain class. Using a classification algorithm and a representative sub-sample of the data it is possible to learn a decision boundary based on the characteristic representation/label pairs, such that the underlying data can be separated into different classes. In this first approach in 2015, the pattern recognition method was used in a forming process for the prediction of the class “crack” for a DX54D steel. This classification approach has shown promise, with a 90% probability of predicting crack initiation, before it occurs. This analysis confirmed the validity of pattern recognition as a suitable tool to analyze material characteristics during Nakajima tests.

Nevertheless, while the class “crack” is easily definable, the onset of instability is challenging. Metallography analyses have shown that patterns related to localized necking are recognizable on the surface during instability for conventional deep drawing steel [13] as well as dual phase steel [14]. Therefore pattern recognition can be potentially used for the determination of forming limits. The consideration of the forming limit as classification problem becomes therefore a point of interest [15]. In the present study, a supervised classification approach using experts’ opinions for the classification of diffuse necking, local necking and crack is presented. In order to qualify the validity of the proposed method for different material behaviors, three materials are investigated: the conventional deep drawing steel DX54D in two different thicknesses, the dual phase steel DP800 and the aluminum alloy AC170. The outcomes from the pattern recognition method are compared to the experts’ annotations and interpreted using the material characteristics. Additionally, multiple FLC candidates are created based on the classification results. These are compared with the conventional and the time-dependent FLC, and with the FLC created by majority voting, based on the experts annotations.

## 2. Experimental Procedure

**Figure 1:**
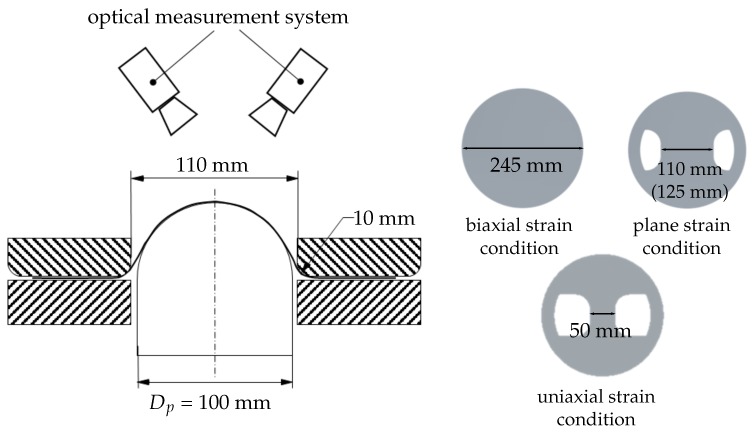
Schematic drawing of the used Nakajima setup and main geometries of investigation.

The used experimental Nakajima setup is depicted in Figure 1. The system is composed by a clamping unit with a inner die diameter of 110 mm, a hemispherical punch with a diameter of 100 mm. The optical measurement system used is ARAMIS from gom GmbH. The two cameras take pictures of the surface from the beginning to the end of the test and the strain distributions are evaluated with the DIC technique. On the specimen surface a layer of white lacquer is applied, in order to minimize the metallic light reflection. Afterwards, a stochastic pattern of graphite is sprayed for the correlation of the pictures.

The investigated different strain paths are obtained by changing the specimen geometry from a full geometry to waisted blanks with a parallel shaft. The width of the remaining material defines the geometry’s name (for example: S050 has a width of 50 mm). Between punch and specimen a lubrication system according to the DIN EN ISO 12004-2 is used. The system is composed by multiple layers of grease, Teflon foil and soft PVC, in order to minimize the friction between punch and specimen and to achieve a crack at the top of the specimen. The frame rate as well as the punch velocity are varied for the different experiments. In order to have different combination of strain paths, materials and boundary conditions, the punch velocity varies from 1 to 2 mm/s and a frequency of 15 Hz, 20 Hz or 40 Hz. As long as the searched class is visible in the video data, the methodology is not dependent on the frame rate or punch velocity.

## 3. Materials and Experts Guidelines

### 3.1. Materials

#### 3.1.1. DX54D

**Figure 2:**
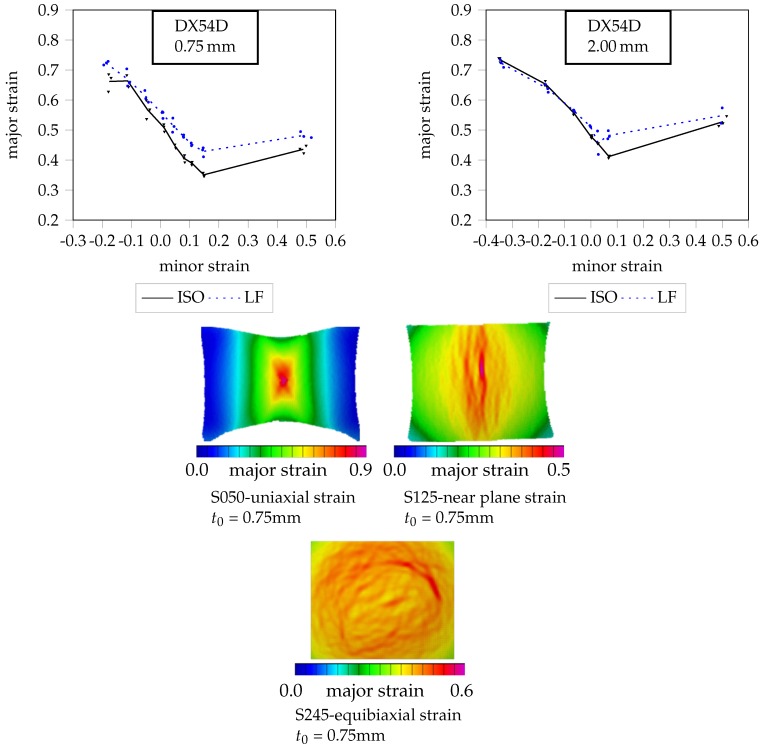
FLC of DX54D (0.75 mm) and DX54D (2.00 mm) with Aramis visualizations of the strain distributions one step before crack.

The DX54D is a deep drawing steel, often used in the automotive industry. It is a ductile metal due to a uniform elongation between 22% and 23% and a relatively low yield strength at 164–170 MPa. Considered a conventional metal, with a tendency for ductile necking, the classic FLC according to the ISO 12004-2 shows a good agreement with the experimental results [8]. In Figure 2, the FLCs as well as the strain distribution prior to crack initiation are shown for uniaxial strain (S050), near plane strain (S125) and biaxial strain (S245) conditions.

According to the DIN EN ISO 12004-2, for the evaluation of the FLC at least 5 points are required. For the DX54D, 9 geometries are considered. On the one hand, more geometries are required to better cover the material behaviour under negative minor strain, since the reached minor strain level by the geometry under uniaxial strain state reaches the high value of 0.3–0.4. On the other hand, the material is ductile and the strain localization is visible several forming steps before crack occurs. Therefore, it is expected that that the necking phase can change between different geometries by the DX54D more than by the high strength steel DP800. Thus, in order to have more data concerning the necking, several geometries have been tested. The FLCs achieved with cross-section and line-fit method are depicted for the two thicknesses. It can be observed that there is a difference between the two FLCs from the cross-section method and the line-fit method. Since the necking transition goes gradually for a few seconds before crack initiation, both methods evaluate the instability point in the necking phase but at different strain levels. The line-fit method is less conservative than the cross-section method with higher values of strain. As it can be observed by the strain distribution before crack, under uniaxial strain the major strain tends to localize at the center, with a typical “x” form strain distribution. Due to the geometry and the anisotropy of the material greater than 2, there is a diffuse necking along the width and the minor strain on the necking zone is negative. The prior slip planes are inclined at about 45° to the rolling direction. Under plane strain condition, the minor strain is near to zero and the prior deformation occurs in the thickness. Nevertheless, a localization on the surface is observable. The biaxial strain condition shows a general diffuse necking, due to the severe thinning. The minor strain value is positive and near to the major strain value and the principal strain along the thickness is two times higher, due to the volume constancy.

#### 3.1.2. DP800

**Figure 3:**
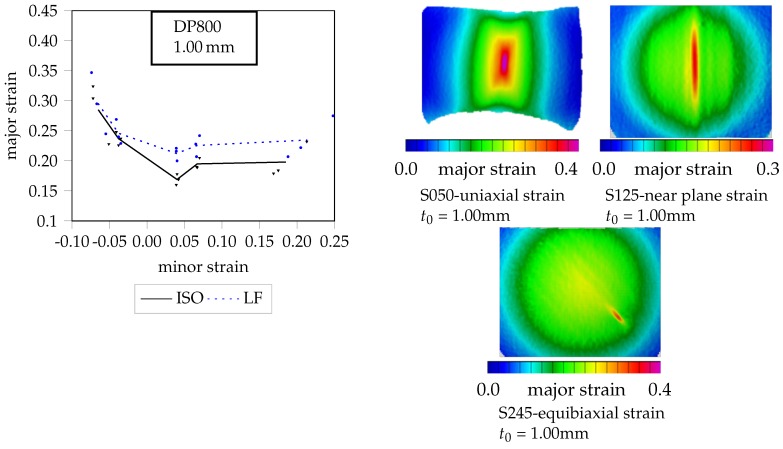
FLC of DP800 with Aramis visualizations of the strain distributions one step before crack.

The DP800 is a dual phase steel with a matrix of ferrite and martensite precipitations. It is a high strength material with a yield strength of 465 MPa and a uniform elongation of 14%–16%. Multiple local maxima are observed during Nakajima tests, due to the inhomogeneous structure of ferrite and martensite [8]. In Figure 3 the major strain distribution before crack initiation is shown. While under uniaxial strain the localization is supported by the specimen geometry, the strain distribution under plane strains shows multiple maxima with a peak at the top of the specimen. Under biaxial stretching the crack initiation occurs without a necking phase but with a sudden localization.

The FLC achieved with the cross-section method has lower values than the line-fit FLC. It can be observed, that the forming limit by biaxial strain condition is low and causes a local peak of the FLC by the geometry S125. this effect can be partially explained with the micro-structure development of the DP800 under this strain condition. As observed in [16], under biaxial strain condition the DP800 show an increase of micro-voids in the thickness during forming that probably causes a reduction of the forming potential. In addition, the deviation of the results shows instabilities for both methods, confirming their weakness by evaluating sheet metal with sudden onset of crack behaviors.

#### 3.1.3. AC170

**Figure 4:**
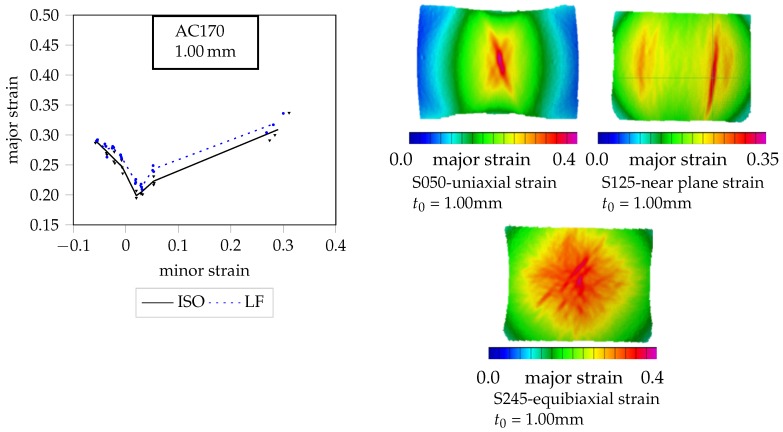
FLC of AC170 with Aramis visualizations of the strain distributions one step before crack.

The AC170 is a light weight aluminum alloy of the 6xxx series. In the T4 condition, it has good formability and it is also used in car-body structures. The yield strength is near to 140 MPa and the uniform elongation is comparable with the DX54D. Nevertheless, this aluminum alloy shows lower formability, in part justified by the low Lankford coefficient. The FLCs achieved by both methods show similar results with a low deviation (see Figure 4). The strain distribution during forming shows a strain localization before crack and therefore more than five geometries are considered. In particular, multiple maxima are observed for the specimen under plane strain. This phenomenon can be related to the material structure of the AA6xx AlSiMg alloy, with several precipitations present in the structure causing local hardening of the material.

### 3.2. Failure Stages and Expert Guidelines

Expert annotations are essential for the use of supervised pattern recognition techniques, for any application. The experts’ knowledge is used in pattern recognition applications for the assignment of different classes to the available data, which is subsequently employed for training the supervised learning algorithm. Further information about the pattern recognition procedure is given in the next section. An example of the use of experts in pattern recognition is given in Matsui et al. [17]. Supervised learning has thus far found widespread use in the fields of computer vision and computational medicine by Jaremenko et al. [18] and Vo et al. [19]. The clinical experience of doctors and specialists develops the recognition skill of anomalies (cancer, musculoskeletal disorder, …) and therefore expert assessment is a suitable method for defining the class of anomaly. In sheet metal forming and in particular by Nakajima test, the anomaly is represented by the onset of necking or, more in general, by any deviation from the homogeneous forming. The variety of forming conditions and material behavior makes a unequivocal definition of classes hard. However, a first evaluation of general classes can be achieved by considering a simple forming condition, like the uniaxial tensile test. The test specimen is subject to a uniaxial force along its length. Due to the relatively small thickness compared to the length and the width, it can be assumed that the stress condition is uniaxial and the principal strain is oriented along the force direction. After a linear increase of the strain with the stress (elastic behavior), the yield point is reached and the material starts to deform plastically. The required stress increases due to material hardening and the strain develops homogeneously. Afterwards the maximal tolerable force is achieved and the material starts to neck. Normally, necking affects the width causing a reduction of the specimen section. Finally, the necking localizes in a small area in the order of the thickness and then the crack occurs. These considerations are used as guidelines for the experts’ annotations. Four classes are defined as follows:

- Homogeneous forming: homogeneous strain distribution on the whole area
- Diffuse necking: inhomogeneity in the strain distribution between the two principal directions; the area of interest is still the whole area
- Local necking: it occurs in a small area in the order of the thickness and a thinning in that area is observed
- Crack: it is defined with the occurrence of damage; surface discontinuities

**Figure 5:**
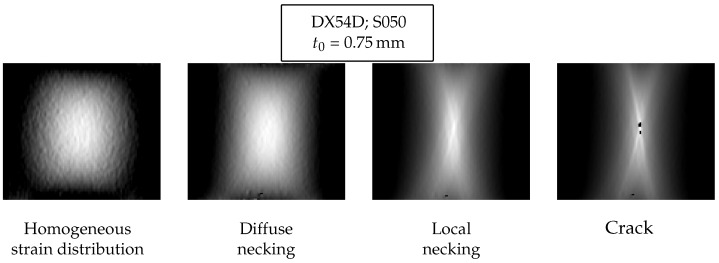
Forming phases guideline for the Nakajima test.

An example of the four classes in Nakajima test is depicted in Figure 5. In the guidelines no other distinctions for the different geometries are made. The defined classes are expected to be compatible for geometries with a small width in the region of negative minor strain, but not for those with positive minor strain. For biaxial straining (full geometry) or plane strain (120–125 mm width) the analyzed surface captured on pictures by the optical measurement system is limited to the free specimen area from the top to the inner diameter of the die (110 mm). Since the diffuse necking occurs in an extended area that covers the whole width, part of the necking area may not be visible and therefore the diffuse necking cannot be recognized. In addition, the different strain conditions cause different strain distribution that deviates from the ideal class definition from the tensile test. Finally, for those materials that do not present a clear transition between uniform elongation and onset of necking, such as high strength material, the definition of the classes can be difficult.

**Figure 6:**
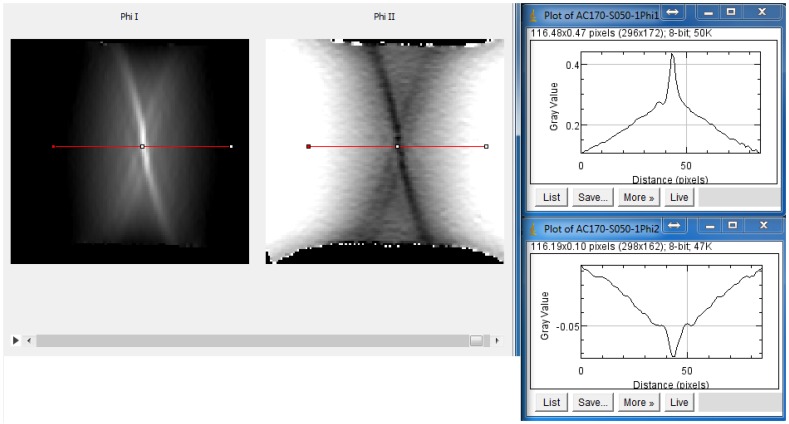
Part of the developed user interface for the collection of experts decisions with on-line cross-sections through the strain distributions.

For the expert decision, the major strain, minor strain and the thickness reduction distributions calculated with the optical measurement system are available as images. The data are accessible using a developed user-interface, as depicted in Figure 6. The timeline can be shifted using a bar and the experts can also define sections on the distribution in order to investigate the strain distribution in 2D dimension. Five experts were interviewed in the present study, since a good correlation is already possible as suggested by Hönig et al. [20]. The experts are scientific researchers in the field of material characterization at the Institute of Manufacturing Technology at the Friedrich-Alexander-University of Erlangen-Nuremberg. They have a minimum of 4 years’ up to 20 years’ experience in material characterization. They are considered experts thanks to their experience with different materials as well as with the usage of optical measurement systems tests were repeated three times for AC170 and DP800, using five different geometries for each material. For DX54D in which a distinct necking phase is recognizable, 9 geometries were tested, in order to detect common necking classes for different widths, with similar behavior.

## 4. Methodology and Machine Classification of Failure Stages

**Figure 7:**
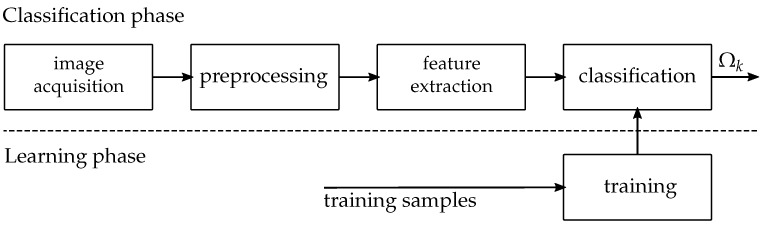
The Pattern Recognition Pipeline: From the original image to the classified result (adapted from [12]).

As described in Section 1, the evaluation of the onset of necking by conventional methods such as the cross-section method or time dependent methods, depends on the evaluation of limited areas or sections that are either based on an arbitrary threshold, require user interaction or focus only on one of the principle strains, such as major strain or thickness reduction. Instead of artificially decreasing the available information on a couple of pixels, the pattern recognition approach exploits the image information of all principle strains at the same time while focusing on the extremal region and its vicinity. To tackle the problem of detecting the three different failure classes, the method follows the conventional pattern recognition pipeline as illustrated in Figure 7. The individual video sequences acquired of different materials and geometries (described in Section 2) serve as inputs to the pipeline. These signals are preprocessed to aid in classification by reducing noise and interpolating missing data resulting from defect pixels. As the method follows the principle of supervised classification, expert knowledge is used to separate the different forming stages into distinct classes. This means each image of a video sequence receives a label from the experts which corresponds to a specific failure class. This procedure leads to a label vector for each sequence, used as ground truth for supervised classification. During *feature extraction* the preprocessed images are transformed into another representation such that other characteristics besides the plain image intensity values can be used to describe the image and thus the current state of failure. This allows the generation of a compressed representation, a characteristic vector, of each image in feature space. The extracted features used in the method are closely related to the experts visual perception and therefore focus on the evaluation of greyscale and edge information or interactions between neighboring pixels and additionally support a comprehensive encoding of strain progression. This data processing sequence is valid for both, the classification and learning phase. To simulate a real-life scenario, that would use a learned classification model, the data set is divided into disjoint training and test sets. The training set is used within learning phase, whereas the test set is used for the simulation of the real-life scenario and thus corresponds to the classification phase. The extracted information, in form of the characteristic and ground truth vectors are used in the *classification* step, wherein a classifier uses the training set to find an optimal solution, that separates instances to different classes. This separation hypothesis is evaluated using the disjoint test set, whereby the class assignments of the individual instances are compared to the corresponding ground truth labels, leading to a class probability $\Omega_{k}$ for each instance of the data. The separation hypothesis is quantitatively evaluated in terms of: the area under receiver operating characteristic curve (ROC) and precision or recall metrics. Subsequent sections describe each component of our classification pipeline in detail.

### 4.1. Preprocessing

The output of the optical measurement system ARAMIS is converted into a three channel (major strain, minor strain, thinning) image which serves as input to the algorithm. The high sampling rate or minor errors during probe preparation may adversely affect the DIC technique, such that it may temporarily be unable to correlate blocks on subsequent images and thus is unable to calculate strain values. This leads to temporal defect pixels rendering it impossible to apply e.g., a convolution with a kernel to extract edge information. As information of this nature is crucial to assess the localized necking state, missing values are interpolated using the temporal progression of individual strain values. In addition to the temporal missing values there exist static defect pixels that deliver no measurement signal during the whole forming procedure. These artifacts are removed by calculating the mean value using a square 3×3 neighborhood. Besides these two types of artifacts defect, pixels may also be a sign of crack initiation, since just before or during material failure the DIC system is not able to further track the individual blocks, which leads to a sudden, increasing amount of defect pixels towards the end of the forming procedure. This indicator has been used by the experts to assign the failure class. As expert annotations are based on this kind of information, removing or interpolating values at these pixels would adversely affect the subsequent classification task. For this reason, defect pixels that do not recover during forming are replaced with a negative value to deliver e.g., a strong edge response that corresponds to material failure and thus crack initiation.

### 4.2. Feature Extraction

In the literature, material failure has been assessed using limited information from cross-sections or small areas, delineated based on an arbitrary threshold defined for one principal strain. Instead of focusing on a subjectively defined area, numerous areas of the available 2D image representations of strains, which include extremal regions and their vicinity. These images are encoded with local descriptors, the so called features, to reduce their dimensionality. The characteristic vector being used by the classification algorithm, is created by concatenating the extracted features of each principle strain, such that information from all three domains can be utilized simultaneously. The selection of suitable features is challenging as this is the first study to use pattern recognition methods to assess multiple stages of material failure. Consequently, we employ features that are considered suitable to capture the continuous progression of strain over time, and have been extensively investigated for various applications in medical image analysis and computer vision.

#### 4.2.1. Histogram

In image processing a histogram represents the frequency of gray level occurrences of the whole image and may provide insight into the characteristics of the image. They do not consider any structural arrangement of pixels or a certain neighborhood and only provide information about the distribution of gray level intensities. The histogram is expected to change between the homogeneous forming phase and the inhomogeneous forming phase as higher intensities occur locally, affecting the skewness and the kurtosis of the distribution. To enable comparison, all histograms computed comprised 256 bins.

#### 4.2.2. Homogeneity

**Figure 8:**
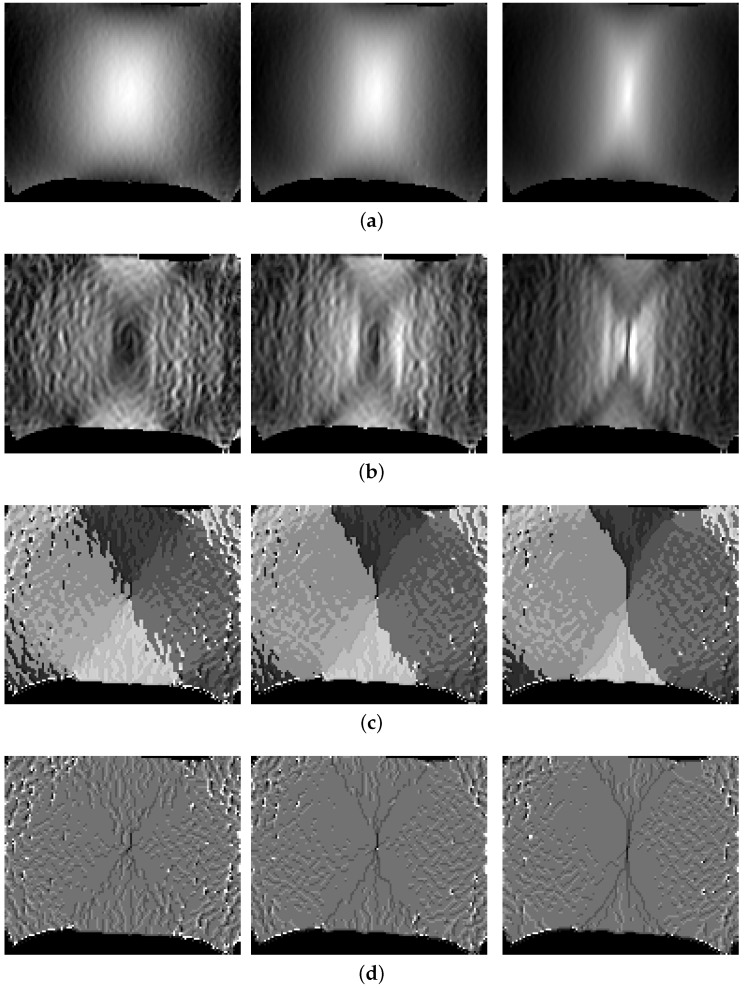
Major strain information in feature space and its progression over time: (**a**) Major strain distribution as greyscale image. (**b**) Sobel filtered gradient information. (**c**) Visualization of uniform LBPs. (**d**) Visualization of rotation invariant uniform LBPs.

Two types of image representations are evaluated using this feature extractor. The first information domain is the original greyscale (strain) distribution and the second information domain consists of the edge information. In the latter case, the intensities are converted into edge representation by convolution with a Sobel operator in x- and y-direction and calculation of their magnitude. Examples of both domains are depicted in Figure 8a,b at different time steps showing the progression of strain and edges. Within both information domains the exact same features are computed. They consist of basic statistical moments up to the fourth order and assess the degree of homogeneity in image appearance. In addition to the statistical moments, the median, the minimum and the maximum values of both domains are considered. To evaluate changes within the structure of the material, the size of the top 10% and top 1% area with respect to the current maximum value of each domain are extracted, in addition to their fraction. Until the end of the homogeneous forming phase, the two areas are expected to progress evenly such that their fraction remains constant. If one of the two areas significantly changes regarding their expansion in size, their fraction will decrease. As these features are extracted for each principal strain, the characteristic vector consists of 60 features.

#### 4.2.3. Local Binary Pattern

Another strategy to describe greyscale distributions are Local Binary Pattern (LBP) developed by Ojala et al. [21]. The advantage of LBP is, that it is able to accommodate intensity variation within images, as one pixel is always evaluated in the context of its nearby neighborhood. Within the original approach by Ojala et al., a 3 × 3 (n=8) local neighborhood is considered to encode the central pixel using a binary weighting scheme. One drawback of LBPs is their rotation dependence, as the comparison of the central-pixel with its neighborhood will change when the image is rotated. Ojala et al. [22] have extended this approach to become rotation invariant, using a circular neighborhood, with the possibility of varying the neighborhood and radius. They introduced uniform LBP (LBPu), as well as rotation-invariant uniform LBP (LBPriu), while the latter are a reduced version of LBPu which contain less information. A visualization of both features is depicted in Figure 8, whereby (c) LBPu encode regions with the same color if the neighborhood with respect to the central-pixel behaves identical and (d) LBPriu highlight the extremal regions of the strain distribution. In the current method the LBPs, as well as an extended version which includes the variance as additional information [23], are extracted with multiple radii and multiple neighborhoods (r=1,3, n=8,16).

#### 4.2.4. Histogram of Oriented Gradients

In contrast to the introduced homogeneity feature, Histogram of Oriented Gradients [24] exploit the gradient orientation, rather than the magnitude of the edges and were introduced in the scope of pedestrian detection. The idea behind the feature extractor is that humans in an upright position can be represented by the orientation of gradients of their outline with dependence on their pose. The degree of invariance to local geometric or human pose changes is addressed with the amount of orientation bins that affect the possible angular resolution. If the angular resolution is too fine, the resulting feature vector would be large and additionally, would result in a fine grained description of e.g., different poses of limbs which is unnecessary for pedestrian detection. In contrast to the original paper, dense sampling was omitted as well as the photometric normalization as the strain distributions are the result of optical measurement, and effects such as variation in illumination or contrast will not occur. Two different angular resolutions (5, 10 degree) are evaluated in a signed (360 degree) an unsigned (180 degree) manner.

### 4.3. Classification Using Random Forests

Random Forests [25] belong to the category of ensemble learners based on the principle of decision trees. Within the training phase, a single decision tree is built with a part of the training data. At each node of the tree, starting from the top, the data is separated, in a binary best split manner based on a threshold value and the evaluation of the characteristic vector of each instance of the data. These comparisons are performed sequentially until no further separation of the data is possible. Within the classification phase, the individual instances of the held out test data are categorized into classes when reaching a leaf node based on the learned comparisons. Using the same input data for growing the classification tree would lead to the exact same classification tree and thus to the same classification result. Random Forest uses multiple strategies to introduce randomness and avoid over-fitting to the underlying data. During training, the data is sub-sampled with replacement and only a random subset of the characteristic vector is evaluated at each splitting node. In addition multiple decision trees are built with individual sub-samples of the training data, leading to different decision trees. Within the classification phase, the remaining instances are categorized based on a majority vote of the learned decision trees.

### 4.4. Experiments

#### 4.4.1. Database

The different failure stages of each material and their distribution is shown in Table 1. Overall, the localized necking and crack class is underrepresented by a large magnitude in all materials in contrast to the homogeneous and diffuse necking class. In general a classifier should be trained in such a way, that it reaches best classification results e.g., best accuracy. Due to the presence of a large imbalance in data (cf. Table 1), a trained classifier would be biased towards the majority class rather than the crack class. On the one hand this can be addressed using a weighting scheme but on the other hand this solution does not increase the variance of the minority classes. To increase the variation of the minority classes data augmentation is used. Each dataset is artificially increased by a factor of 12, using vertical and horizontal flipping as well as small random rotations (2–12 degree). Besides increasing the variation of the dataset, the random rotations also address slight differences regarding the orientation of investigated probes that might occur during preparation. Overall, this procedure increases the data imbalance regarding the amount of instances per class. To address this imbalance, the data is randomly sub-sampled or up-sampled with respect to the amount of instances (50%) of the augmented localized necking class. In this way the variance of each class is increased and a uniformly distributed amount of data can be used for classification.

#### 4.4.2. Inter-Rater-Reliability

The consistency among the annotations of the five raters is examined using the Fleiss’ kappa metric [26]. This is a robust measure for agreement which considers the possibility of the agreement occurring by chance between multiple raters. Using this strategy it is possible to examine how consistent human experts are among each other in terms of their categorization into different failure stages. A value below 0 would correspond to no agreement, while a value between 0.41–0.61 corresponds to moderate agreement and 0.81–1.00 would be considered a perfect agreement.

#### 4.4.3. Classification

To evaluate the performance of the classification algorithm, a Leave-One-Sequence-Out cross-validation (LOSO-CV) setup is carried out, while the majority vote over the experts’ annotations serve as ground truth. Furthermore to discount the influence of different geometries and materials, the LOSO-CV is evaluated geometry-wise for each material. In this way, the classifier is retrained three times within each geometry, such that two-thirds of the data are for training, while the left-out data set is used to asses the separation hypothesis. The number of decision trees used in the random forest classifier is fixed at 200, while suitable values for two other parameters, the maximum depth per tree as well as the minimum amount of samples per leaf are found via grid-search (15–30, 2–12, respectively) per trial of each geometry, in order to improve the generalization of the classifier. To address the unbalanced occurrence of uniaxial, biaxial and plane strain geometries and to generate an unbiased evaluation, the performance of the different features is assessed in two ways. The uniaxial to plane strain images are jointly evaluated using the S060, S080 and S100 geometry, or S050 and S110 if S080 and S100 are unavailable, as the images as well as the necking development appear comparable. The S245 geometry is evaluated independently, as the strain distribution appears very different in this geometry in comparison to the uniaxial to plane strain distributions and thus might require another evaluation area as well as feature for good separation. During training of the classifier, no particular class is preferred over the other, meaning that a miss-classification of the crack class or diffuse necking class has the same cost and thus no weighting scheme is used. Consequently, the general performance and quality of the four class classification problem is evaluated with the average area under ROC curve (AUC), which describes the capability of the classifier respecting all false positive rate thresholds without the need of choosing the best operating point, usually defined based on costs. This facilitates a comparison between the different features as only one value for each four class classification problem has to be considered. The individual best performing features of each material is then evaluated on the respective unrestricted dataset to asses the overall performance of the classification algorithm. In this case, the method is analyzed with a confusion matrix that corresponds to the 50% probability threshold and defines the class membership, such that the performance per class can be investigated in terms of miss-classifications. This allows class-wise assessment in terms of precision and recall.

**Figure 9:**
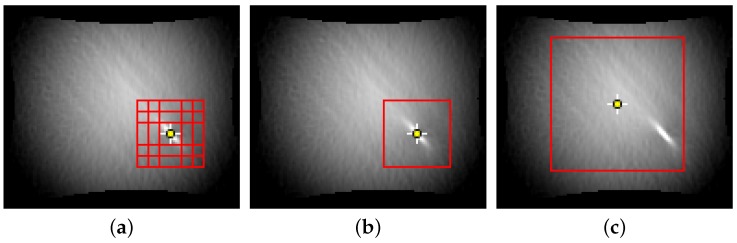
Different evaluation areas of S245-equibiax. (**a**) Multiple patches (9 × 24) are distributed around the maximum with an overlap of 50 % (Patch-wise). (**b**) Evaluation area of one single patch (1 × 36) covering the same area as the multiple smaller patches (Single-patch). (**c**) One large centered evaluation patch covering most of the image (Centered).

Another aspect that needs clarification is the definition of an evaluation area. On the one hand it might be beneficial to focus only on local image details, but on the other hand there might be unused information in the surrounding area that may improve the classification process. For this reason three different patch extraction techniques are investigated that are visualized for DP800-S245 in Figure 9. The first approach extracts multiple patches with an overlap of 50% with a side-length of 16–32 px and a step-size of 8 px, while the maximum value lays within the center (Patch-wise). The second approach covers the same area as the first one with only one patch that leads to a side-length of 24, 36 and 48 px (Single-patch). The third approach covers as much information as possible depending on the underlying sample geometry. As the size of the image may change over time, the evaluation area is defined on the last image without a defect pixel. In contrast to the other two approaches it is not possible to use the maximum value as center pixel as it will lead to image patches that include non-comparable image information e.g., imagine the maximum value lying next to the image border in one sequence and close to center within another sequence. This would lead to a comparison between patches containing unequal information. For this reason the center of mass is used as center of the evaluation area (Centered). A direct comparison of the patch-wise approach with the other strategies is not possible, as each of the nine patches is classified with its own individual probability. To allow a comparison the average image probability is calculated as the mean probability over the individual patches.

## 5. Results

### 5.1. Inter-Rater-Reliability

**Figure 10:**
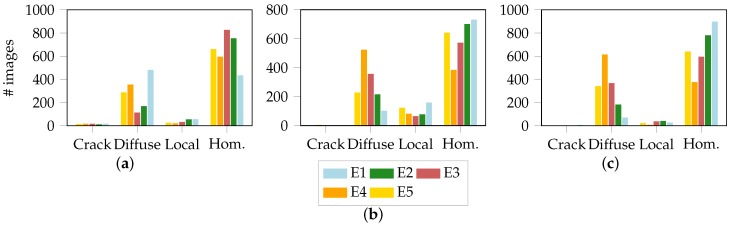
Marginal expert distributions per geometry of DP800: (**a**) S050-uniaxial strain. (**b**) S110-near plane strain. (**c**) S245-equibiaxial strain.

Upon examination, expert annotations were found to be rather diverse and led to different consistencies depending on the material and geometry. The marginal distributions of the individual experts exemplified by S050, S110 and S245 of DP800 are depicted in Figure 10. Each diagram combines the annotations of three different trials for the S050, S110 and S245 geometry, respectively. Especially the separation between the homogeneous and diffuse necking class seems problematic while the experts remain more consistent regarding the crack and localized necking class.

The quality of consent among the individual raters of the remaining geometries and materials are summarized in Table 2. Highest consistency is achieved with a substantial agreement in case of the geometries < S125 of DX54D (2.00 mm), a material that was recorded with 20 Hz sampling rate. A moderate agreement is present in case of DP800, once again the best results are obtained in geometries < S125, while the sampling rate was 40 Hz during the forming experiments. Within AC170 (15 Hz sampling rate) a rather moderate agreement is achieved independent of the underlying geometry, while in the case of DX54D (0.75 mm) (40 Hz sampling rate) consistency among experts drops to poor agreement, in addition no clear trend is observable.

### 5.2. Classification

**Figure 11:**
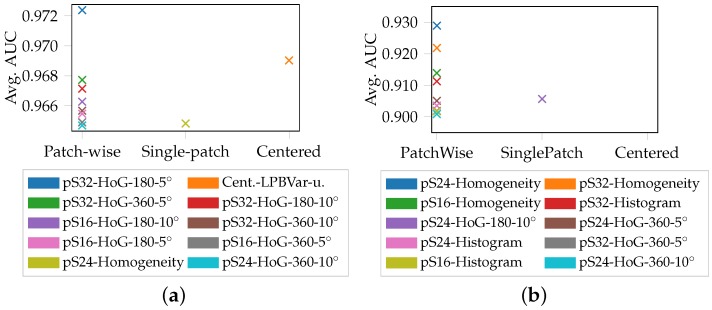

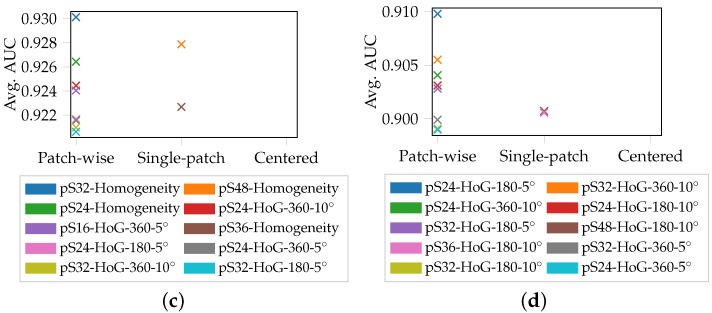
Average AUC calculated over S060, S080/S050-uniaxial strain and S110/S100-near plane strain: (**a**) DX54D (2.00 mm). (**b**) DP800. (**c**) DX54D (0.75 mm). (**d**) AC170.

The ten best results for the uniaxial to plane strain geometries (S060, S050/S080 and S100/S110) of the individual materials are depicted in Figure 11, where pS./Cent. denotes the size of the evaluation area, 360/180 the signed/unsigned Hog. mode and 5°, 10° the resolution of the Hog. in degrees. The average area under ROC curve of the different evaluation approaches are compared, with respect to the underlying feature employed. Overall features that exploit edge information like Hog and Homogeneity dominate the results and in all materials an average AUC above 0.9 can be reached. Only in case of the ductile DX54D (2.00 mm), LBPs coupled with variance perform comparably (0.97 AUC). A trend towards the patch-wise evaluation strategy emerges as the best performance of all materials are from this category. Within DX54D (2.00 mm) and DX54D (0.75 mm) the differences between patch-wise, single-patch and centered are insignificant, whereas in case of DP800 the patch-wise method clearly outperforms the other approaches (0.93 AUC vs. 0.91 AUC). The top ten results of DX54D (0.75 mm) are primarily patch-wise, whereas once again the differences between patch sizes and classification results are insignificant such that pS32-Homogeneity (0.93 AUC), pS48-Homogeneity (0.928 AUC) and pS24-Homogeneity (0.927 AUC) can be considered on par.

**Figure 12:**
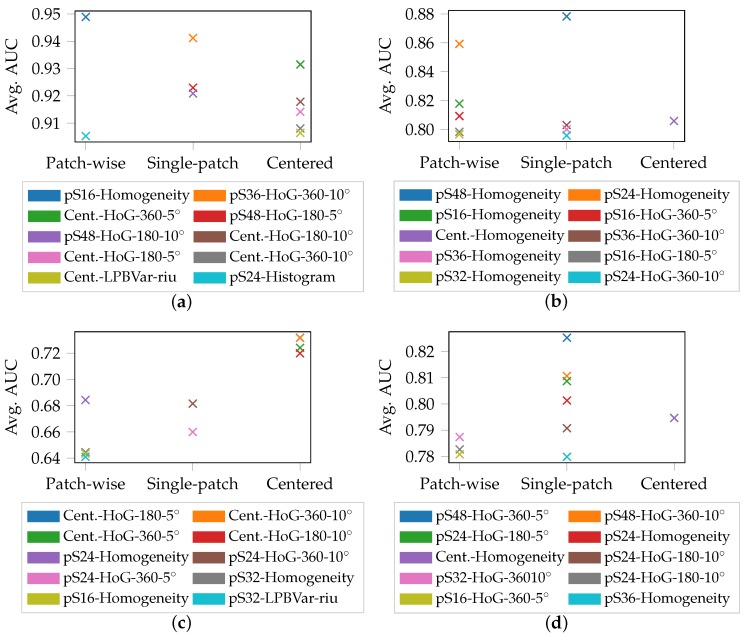
Average AUC of S245-equibiaxial strain: (**a**) DX54D (2.00 mm). (**b**) DP800. (**c**) DX54D (0.75 mm). (**d**) AC170.

The evaluation of the biaxial S245 geometry draws a different picture as visualized in Figure 12. In case of DX54D (2.00 mm) and DP800 very good results with an AUC of 0.95 and 0.88 are reached, outperforming DX54D (0.75 mm) and AC170 with an AUC of 0.725 and 0.825, respectively. In none of the materials it is possible to obtain the best results with an LBP based feature extractor and consequently inferring that edge information is more descriptive and relevant to evaluate, for the classification task. This is emphasized by the fact that superior results are achieved with either Homogeneity or Hog features. Additionally, these results suggest that there is a clear advantage to employing larger evaluation areas. Only within DX54D (2.00 mm) the patch-wise approach slightly outperforms the single-patch and centered strategy.

The AUC condensed the multi-class classification results into one performance indicator and thus facilitates the progress of finding the best performing feature per material. As the uniaxial to plane strain geometries dominate the data distribution, the best performing feature for every material is selected individually for the overall classification experiment. Within all but DX54D (2.00 mm) the best performing feature of the uniaxial to plane strain experiment is at least in the top ten when it comes to S245 geometry and thus only affects the overall result slightly negatively. For this reason the following material/feature pairs are evaluated:

- DX54D (2.00 mm)/pS32-HoG-180-5°
- DP800/pS24-Homogeneity
- DX54D (0.75 mm)/pS32-Homogeneity
- AC170/pS24-HoG-180-5°

In contrast to the above experiments, the per class performance is emphasized by the confusion matrices in Table 3 in terms of classification errors, precision and recall. Each row of the confusion matrix stores the relative frequency of instances in the expected failure categories, whereas the columns contain the relative frequency of instances of predictions made by the random forest. With a perfect agreement between the classifier and the experts, only the diagonal elements of the confusion matrices would contain non-zero entries. Based on this per class evaluation it is possible to conclude which classes are accurately classified by the classifier and those that are not. Overall, all matrices and materials share the similarity that the off diagonal miss-classifications occur during transition between successive classes (i.e., transition between failure stages). Most of the errors are committed between C0 and C1 especially in case of DP800, DX54D (0.75 mm) and AC170 as the C0/C1 and C1/C0 confusions add up to >29%, whereas within DX54D (2.00 mm) the error is <10%. The error between C1 and C2 is considerably lower among all materials as the confusions sum up to <20%. The least error is committed between C2 and C3 as it consistently stays below 20%, whereby in this case the result is influenced by the low support of instances and thus one miss-classification contributes more to the error with respect to the other classes. Best results among all failure classes with the exception of the crack class are achieved within DX54D (2.00 mm) with a recall above 92% mainly due to the low sampling frequency and good inter-rater-reliability. The diffuse necking is classified with the least reliability in case of DP800 and AC170 as the C1 recall corresponds to 69% and 79%, respectively. When comparing the recall of the diffuse necking class of DX54D (2.00 mm) and DX54D (0.75 mm) it is noticeable that the recall in case of DX54D (2 mm) is remarkably higher in comparison with DX54D (0.75 mm), 95% to 84%, what might again be an effect of the sampling rate differences. The same effect is visible in case of the localized necking class as a higher recall with 92% is achieved within AC170 and DX54D (2.00 mm) and only 85% in case of DP800 and DX54D (0.75 mm) that were recorded with a higher sampling rate.

Based on the absolute amount of confusions between class transitions the expected average deviation in terms of images and mm punch movement per sequence is approximated by summing up the miss-classifications and dividing it with the quantity of underlying sample geometries and trials (cf. Table 4). As before, the largest deviation occurs between homogeneous and diffuse necking class, whereas the deviation within DP800 and DX54D (0.75 mm) is comparable, each with >40 images and >1 mm punch movement. AC170 exhibits the lowest deviation with 6 images and 0.6 mm punch movement, while DX54D (2.00 mm) reveals the largest deviation with 20.7 images and 1.66 mm punch movement. Considering the confusion between diffuse necking and localized necking this deviation decreases remarkably to 7.8/2.2 images and 0.2/0.22 mm punch movement for DP800/AC170. In case of DX54D (2.00 mm) and DX54D (0.75 mm) the deviation is identical as both exhibit a deviation of 0.29 mm punch movement which corresponds to 3.9 and 11.4 images, respectively. The lowest error among assessed based on class confusions, is between localized necking and crack class with a deviation of <1 images or <0.1 mm punch movement.

**Figure 13:**
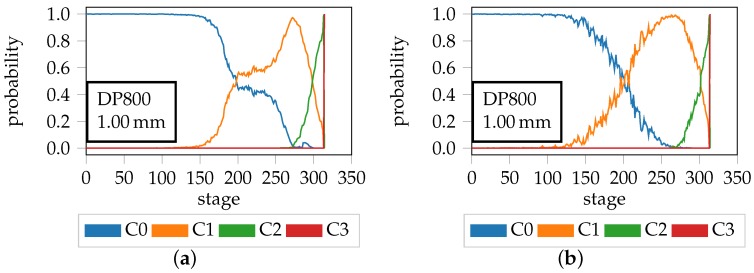
Probability progression over time of DP800-S060-2 of (**a**) PS 24 Homogeneity and (**b**) PS 24 HoG 180 10°.

Apart from these quantitative results a more qualitative interpretation is possible, which takes into consideration the time or stage of the forming procedure. Figure 13 depicts the class probability of belonging to one of the four classes per time-step, for two different features of the same forming trial. The left hand side shows the result of the best performing PS24-Homogeneity feature, whereas the right hand side depicts the result of the slightly worse performing PS24-HoG-180 feature. The region of interest in both images are the transition areas between the individual classes. Especially in case of Homogeneity C0 and C1 are separable, as there exists a clear cutoff point, whereas in case of the Hog. feature this separation is not as distinctive as the C0 and C1 curves oscillate. This renders the Homogeneity feature more appropriate to separate the homogeneous from the diffuse necking class, which is understandable as during the transition from C0 to C1 no orientated edge information is expected to occur. Considering the transition from C1 to C2 both features are appropriate, as a smooth separation with no oscillating behavior evident, and the steepness of the curve indicates that the Hog feature achieves a more reliable separation in this case. This emphasizes that edge orientation coupled with the magnitude and the orientation free edge information coupled with grey-scale information, are suitable to distinguish diffuse necking from localized necking. Consequently, both features are deemed appropriate to separate the localized necking from the crack class as depicted by the sudden and steep transition between C2 and C3.

### 5.3. Comparison Experts vs. Machine

The oscillating behavior of some features within the class transition areas cause difficulties in finding the appropriate point in time that can be used as a lookup for strain values to create the forming limit curves. If the classifier provides a oscillating result vector e.g., $\hat{y}=00010101111222223$, where 0,1,2,3 denote the classes from homogeneous to crack and their positions correspond to the point in time, it is inconclusive when the transition from C0 to C1 takes place as the probabilities vary around 50%. This fluctuation is addressed by using only the connected class probabilities as belonging to one class, this means the result vector would be interpreted as $\hat{y}=00000001111222223$, while the first occurrence of each class is used as the time-step to lookup the corresponding principle strain values. To be comparable to the line-fit method, the same definition of the localization area is used to ascertain whether the point in time that is used as lookup is responsible for the deviation. As a comparison with the expert decisions is pursued, the same lookup strategy regarding the point in time and the evaluation area is used for the expert annotation vector, which of course does not exhibit a oscillating behavior.

**Figure 14:**
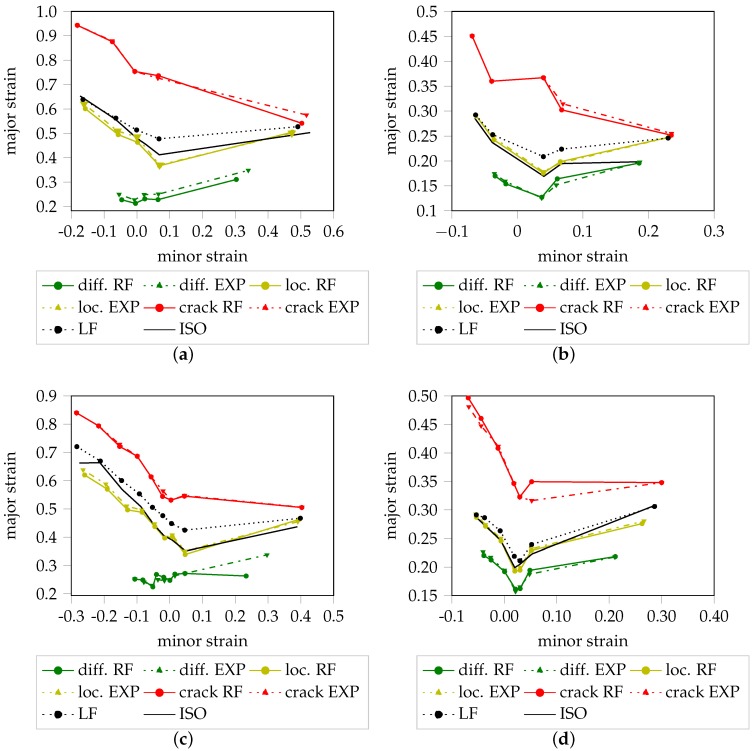
Comparison between expert annotations and machine learning algorithm using all geometries: (**a**) DX54D (2.00 mm). (**b**) DP800. (**c**) DX54D (0.75 mm). (**d**) AC170.

A graphical representation consisting of three failure stages for each forming limit candidate and material is depicted in Figure 14. Every failure class of the classification result is compared to the expert ratings, where, the solid lines represent the result of the classifier and the dashed lines denote the annotated result of the experts. Additionally the standard ISO method as well as the line-fit method are included for comparison. Within DX54D (2.00 mm) and AC170 the diffuse necking curve of the classifier and the experts’ annotation are highly correlated over all evaluated geometries with only a slight deviation regarding the principle strains. With the exception of S125, this holds true for DP800 as well. In the case of DX54D (0.75 mm) the situation is different as the classification results oscillate around the expert results with a clear discrepancy for S245. This is likely due to the poor agreement among experts as evidenced by the low Fleiss’ kappa values in Table 2. As the majority vote is used to train the classifier a poor agreement likely leads to majority votes per trial that are inconsistent with each other and thus correspond to failure stages that are less comparable in terms of their occurrence in time and forming progression, and with respect to their strain distributions. The localized necking curve of the experts as well as the classified result are in accordance with each other, independent of the underlying sample geometry and evaluated material, and exhibit only slight deviations for S050 and S060 within DX54D (0.75 mm). Overall the expert generated curves as well as the curves of the classification approach are comparable to the DIN EN ISO 12004-2 generated forming limit curve rather than the one generated using the line-fit method. This is emphasized within DP800 and AC170 and is particularly pronounced among the uniaxial to plane strain geometries. In all materials with the exception of AC170 the curve representing the crack class of the classifier and the experts’ annotation are on par. The rather large deviation in the case of AC170 results from the low sampling rate. Consequently, there are large deviations in the strain distribution from one time step to the next. Additionally, mis-labeled instances (generated by the experts) may contribute to the observed deviations as well. The latter is supported by the fact that not all experts consistently labeled defect pixels as being representative of the initiation of the crack class. Another observation is the small gap between the localized necking class and initiation of the crack class in the case of DX54D (2.00 mm) and DP800. This permits very little additional forming, prior to failure of the sample. In contrast, this gap is rather large for the other two materials, and in the case of AC170, may be caused by the low sampling rate.

## 6. Discussion

The determination of necking using pattern recognition requires a clear definition of failure classes. For this reason, in the present study, a novel approach for categorizing forming processes into multiple failure classes based on pattern recognition has been presented. The strain distribution during the Nakajima tests was measured using digital image correlation (DIC) with an optical measurement system. The strain distribution characterized by the major strain, minor strain and thickness reduction was used by experts to visually assess forming sequences.

The expert annotations highlighted the heterogeneity of necking behavior for the different materials and strain conditions. While the crack class is well defined and easily recognizable, the diffuse and local necking can be differently interpreted. Focusing on the difference between the strain distribution (Figure 10), it can be observed, that for the local and diffuse necking classes the experts’ knowledge shows low deviation for the geometry S050, while the diffuse necking class strongly deviates for the S110 and S245 geometries, confirming the heterogeneous material behavior under different strain condition. The geometry S050 is under a near uniaxial strain behavior. The negative minor strain and the diffuse necking along the width could be well evaluated from the experts. The test specimens under near plane strain (S110) and the specimens under biaxial condition (S245) show discrepancies between the experts decision regarding the diffuse necking. In addition, the number of images of homogeneous strain distribution are more than the stages in which an instability is observed. This demonstrates that the onset of instability of Nakajima tests occurs few millimeters of punch displacement before crack initiation, according to the definition of instability by the line-fit method [9]. The most challenging geometry is represented by the S245 specimen under biaxial stretching. The biaxial stretching affects the whole evaluation area with a homogeneous thinning. Independently from the material, the strain distribution develops gradually causing different pronounced degrees of severe thinning until a crack occurs. Therefore, the experts have recognized a local necking only a few stages prior to crack initiation, while diffuse necking is considered to start at very early stages. It has also to be noted, that the guidelines used by experts for the general definition of diffuse necking and local necking were based on the tensile test. In this simple test, the stress can be considered uniaxial and it can be easily described by considering only the planar components. However, in Nakajima test with higher widths, the stress conditions are more complex. Therefore, while for small sample width in which the strain distribution is similar to the uniaxial tensile test, the general definition of necking is well defined and easy to detect, with wider samples the stress conditions cause more complex strain developments which are not directly detectable as general diffuse and local necking.

Also the material structure seems to play an important role, even if the general consideration about the differences due to different strain paths are valid for all investigated materials. Based on Figure 10 depicting the experts’ decisions for the geometry under near plane strain condition for the investigated material, it can be observed that the diffuse and local necking classes were differently interpreted by the experts for the different materials. This is also emphasized by the Fleiss-Kappa values in Table 2 as the quality of agreement decreases starting from S110 while reaching its minimum at S245. This degeneration is mainly introduced due to the low consistency of the homogeneous and diffuse necking classes as they dominate the forming process and thus negatively affect the measure. Additionally, it might also be a side effect of the localized necking behavior as it is not focusing on a small area but occurs distributed over larger parts of the image and thus renders it more difficult for experts to find the exact point in time, when the onset of necking takes place. This phenomenon also explains the consistently low Fleiss-Kappa values of DX54D (0.75 mm) as the ductility of the material coupled with a high frequency intensifies the problem for the experts to distinguish homogeneous state from diffuse necking, and diffuse necking from localized necking. In contrast, when using a low sampling rate (AC170 and DX54D (2.00 mm)) or analyzing a less-ductile material (DP800) a moderate to good agreement between the experts is achieved. Figure 10 emphasizes that the annotations for localized necking are consistent among experts even in the case of a low kappa-value for the specimen under biaxial loading (S245). The consistently good agreement within the localized necking class might be the consequence of the graphical user interface that supported the rater with the possibility of drawing virtual cross sections along the specimen reducing the variance between the experts.

Overall, the classification results (cf. Figure 3) emphasize that coupling expert knowledge with a classification algorithm, is a suitable approach to assess the failure behavior of sheet metals. This is particularly true for the detection of localized necking, as the ability of the experts to differentiate between different failure states by visual inspection, was a valuable source of information, as highlighted by the 85% recall for all materials. As the classification algorithm is influenced by the ground truth of the rater majority, the low consistency among experts and thus the high sampling rate also affects the classification performance. Inconsistent annotation of sequences thus lead to higher miss-classifications in the transition areas from one class to the other. This is highlighted by the lower recall for diffuse necking in comparison to the localized necking in all materials with the exception of DX54D (2.00 mm). Of course this under-performance might also be a limitation of the chosen feature space, as they are focusing on edge information that seems better suited to the localized necking state, but the inconsistency between experts renders this impossible to evaluate with certainty.

Three different evaluation strategies were investigated. The patch-wise strategy turned out to be the superior approach when evaluating uniaxial to plane strain geometries with a patch size of 24 px or 32 px using Homogeneity or HoG features. In contrast, for the biaxial condition S245 a larger evaluation area was found to be beneficial, using either a single-patch that is centered around the maximum of the last valid stage or based on the centered approach that uses the point of mass. For optimal classification results the evaluation strategy would have to be applied separately depending on the specimen geometry, but as the the uniaxial geometries dominate the data distribution only the best performing features were investigated. Additionally it is more important to have consistent and precise annotations regarding the point in time when necking occurs rather than the choice of feature.

The forming limit curves of the experts coincide very well with the candidates of the classification algorithm (cf. Figure 14). This is particularly true for the localized necking candidate, which exhibits a high correlation. This is reasonable as the decision of the experts for this class were made using the graphical user interface that supported the rater with on-line virtual sections along the strain distribution and thus e.g., a sudden increase in the strain distribution could easily be detected, which might also explain the good agreement with the ISO determined forming limit curve. Additionally, this observation explains why the Homogeneity feature or HoG outperform the LBP features, as the latter are not able to capture rising intensity differences without being extended by variance as only binary comparisons are possible. Despite the encouraging results achieved in this study, the proposed method still has several limitations: (1) one has to decide to use a specific feature in advance or try all of the possible features rendering the method time consuming; (2) selection of the evaluation area is ambiguous, while for uniaxial geometries patch-wise evaluation seems appropriate, biaxial geometries require a large evaluation area, for best results a mixed evaluation strategy would be reasonable; (3) geometry wise evaluation and restriction to one material excludes side effects that might be beneficial for the classification process as the data is very imbalanced and limited with this setup, which was partly addressed with augmentation; (4) generation of ground truth annotations is expensive as multiple experts need to be consulted to asses the failure behavior based on visual inspection for every new material investigated; (5) lack of consistency between experts’ annotations for the diffuse necking class. The main drawback of the presented method is the dependence on expert knowledge (4), that could be addressed using unsupervised classification algorithms which focus only on the localized necking criteria and thus omit the low consistency of the diffuse necking class. If the experts would be able to define a consistent point in time within the strain distributions, the pattern recognition system would easily be able to separate classes, as within feature space the trials of each geometry are comparable. The classification performance is thus limited to the expected error of the experts and therefore an improvement of the ground truth, maybe based on metallographic examinations, should be pursued. Metallography investigations on the DX54D [13] and on the DP800 [14] suggested an improvement of consistency by using metallography outcomes with a better understanding of the material behavior during Nakajima tests. Despite these limitations, the presented work highlights the potential of conventional pattern recognition methods and allows staging the failure behavior of sheet metals based on image information. In particular, it has been shown that experts are able to assess the failure status of specimen during sheet metal forming processes, that their knowledge can be transfered and used to create a forming limit curve that consists of multiple failure stages, without the restriction of a limited evaluation area and the necessity to decide on a distinct principle strain. This study enables future work on other materials with more complex necking behavior and highlights the applicability of pattern recognition methods.

## Figures and Tables

**Table 1:** Amount of available images per failure class–sum over all geometries per material.

Material	Incon.	Diff. Neck.	Loc. Neck.	Crack	Total
DX54D (2.00 mm)	5303	1246	273	35	6857
AC170	435	430	195	30	1090
DP800	3156	1335	292	45	4828
DX54D (0.75 mm)	2802	4953	773	220	8748

**Table 2:** Inter-Rater-Reliability in terms of Fleiss-Kappa statistics.

Geometry	DX54D (2.00 mm)	DP800	DX54D (0.75 mm)	AC170
S050	–	0.56	0.27	0.39
S060	0.70	0.59	0.24	0.38
S070	–	–	0.22	–
S080	0.70	–	0.30	0.52
S090	–	–	0.24	–
S100	0.70	–	0.33	0.51
S110	–	0.56	0.34	0.43
S125	0.38	0.38	0.24	0.37
S245	0.33	0.35	0.05	0.41

**Table 3:**
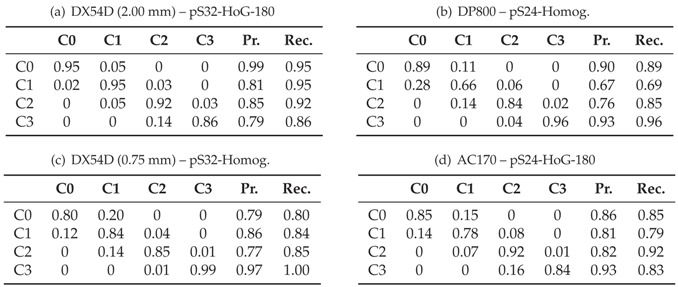
Overall confusion matrices of the best performing feature for each material.

**Table 4:** Average error per sequence and class (in ± # images/± mm punch movement).

	C0/C1	C1/C2	C2/C3
DX54D (2.00 mm)	20.7/1.55	3.9/0.29	0.9/0.07
DP800	46.3/1.15	7.8/0.20	0.3/0.01
DX54D (0.75 mm)	42.0/1.05	11.4/0.29	0.25/0.01
AC170	6.0/0.60	2.2/0.22	0.3/0.03

## References

[1] Col A. FLCs: Past, present and future. Proceedings of the Iddrg 2002.

[2] DIN Deutsches Institut für Normung e.V. (2008). Metallische Werkstoffe –Bleche und Bänder-Bestimmung der Grenzformänderungskurve–Teil 2: Bestimmung von Grenzformänderungskurven im Labor.

[3] Nakajima K., Kikuma T., Hasuka K. (1968). Study of Formability of Steel Sheets.

[4] Marciniak Z. (1965). Stability of Plastics Shells under Tension with Kinematic Boundary Condition. Arch. Mech..

[5] Bragard A., Baret J.C., Bonnarens H. (1972). Simplified Technique to Determine the FLD on the Onset of Necking. C. R. M..

[6] Chu T.C., Ranson W.F., Sutton M.A., Peters W.H. (1985). Applications of digital-image-correlation techniques to experimental mechanics. Exp. Mech..

[7] Lewison D.J., Lee D. Assessment of Experimental Methods for Determination of Forming Limits. Proceedings of the NUMISHEET 1999.

[8] Merklein M., Affronti E., Volk W., Jocham D. (2017). Verbesserung der zeitlichen Auswertemethoden von Versuchen zur Ermittlung der Grenzformänderung und Ableitung eines virtuellen Ersatzmodells.

[9] Volk W., Hora P. (2011). New Algorithm for a robust user-independent evaluation of beginning instability for the experimental FLC determination. Int. J. Mater. Form..

[10] Merklein M., Kuppert A., Mütze S., Geffer A. New Time dependent method for determination of FLC applied to SZBS800. Proceedings of the 50th IDDRG 2010.

[11] Merklein M., Maier A., Kinnstätter D., Jaremenko C., Affronti E. (2015). A New Approach to the Evaluation of Forming Limits in Sheet Metal Forming. Key Eng. Mater..

[12] Niemann H. (1983). Klassifikation von Mustern.

[13] Affronti E., Merklein M. (2017). Metallographic Analysis of Nakajima Tests for the Evaluation of the Failure Developments. Procedia Eng..

[14] Affronti E., Weidinger M., Merklein M. Metallographic analysis of failure mechanisms during Nakajima tests for the evaluation of forming limits on a dual-phase steel. Proceedings of the IDDRG 2018.

[15] Jaremenko C., Huang X., Affronti E., Merklein M., Maier A. Sheet metal forming limits as calssification problem. Proceedings of the 15th IAPR International Conference on Machine Vision Applications (Machine Vision Applications).

[16] Tasan C.C., Hoefnagels J.P.M., Horn C.T.H., Geers M.G.D. (2009). Experimental analysis of strain path dependent ductile damage mechanics and forming limits. Mech. Mater..

[17] Matsui K., Kawaguchi K. (2015). The Differences of Subjective findings in Pattern Recognition among Experts, Novices and Students: A Quasi-Delphi Technique. Educ. Med. J..

[18] Jaremenko C., Maier A., Steidl S., Hornegger J., Oetter N.K.C., Stelzle F., Neumann H. (2015). Classification of Confocal Laser Endomicroscopic Images of the Oral Cavity to Distinguish Pathological from Healthy Tissue. Bildverarbeitung für die Medizin 2015.

[19] Vo K., Jaremenko C., Bohr C., Neumann H., Maier A. (2017). Automatic Classification and Pathological Staging of Confocal Laser Endomicroscopic Images of the Vocal Cords. Bildverarbeitung für die Medizin 2017.

[20] Hönig F., Batliner A., Nöth E. How Many Labellers Revisited–Naïves, Experts, and Real Experts. Proceedings of the Speech and Language Technology in Education.

[21] Ojala T., Pietikäinen M., Harwood D. (1996). A comparative study of texture measures with classification based on featured distributions. Pattern Recognit..

[22] Ojala T., Pietikainen M., Maenpaa T. (2002). Multiresolution gray-scale and rotation invariant texture classification with local binary patterns. IEEE Trans. Pattern Anal. Mach. Intell..

[23] Guo Z., Zhang L., Zhang D. (2010). Rotation invariant texture classification using LBP variance (LBPV) with global matching. Pattern Recognit..

[24] Dalal N., Triggs B. Histograms of Oriented Gradients for Human Detection. Proceedings of the 2005 IEEE Computer Society Conference on Computer Vision and Pattern Recognition (CVPR’05).

[25] Breiman L. (2001). Random forests. Mach. Learn..

[26] Fleiss J.L., Cohen J. (1973). The Equivalence of Weighted Kappa and the Intraclass Correlation Coefficient as Measures of Reliability. Educ. Psychol. Meas..

